# An Innovative Chemical Adherence Test Demonstrates Very High Rates of Nonadherence to Oral Cardio-Metabolic Medications

**DOI:** 10.1016/j.ekir.2023.09.033

**Published:** 2023-09-29

**Authors:** Hanad Osman, Dan Lane, Dennis Bernieh, Samuel Seidu, Prashanth Patel, Kamlesh Khunti, Nigel J. Brunskill, Gang Xu, Pankaj Gupta

**Affiliations:** 1 Diabetes Research Centre, Department of Population Health Sciences, College of Life Sciences, University of Leicester, Leicester, UK; 2 Department of Metabolic Medicine and Chemical Pathology University Hospitals of Leicester NHS Trust, Leicester, UK; 3 Leicester van Geest Multi-Omics Facility, Hodgkin Building, University of Leicester, Leicester, UK; 4 Department of Cardiovascular Sciences, University of Leicester, Leicester, UK; 5 National Institute for Health Research (NIHR) Leicester Biomedical Research Centre, Glenfield Hospital, Groby Road, Leicester, UK; 6 NIHR Applied Research Collaboration East Midlands, Leicester General Hospital, Leicester, UK; 7 John Walls Renal Unit, Leicester General Hospital, University Hospitals of Leicester NHS Trust, Leicester, UK

**Keywords:** cardio-metabolic disease, cardio-metabolic medications, chemical adherence testing, chronic kidney disease, nonadherence

## Introduction

Chronic kidney disease (CKD) is one of the leading causes of death worldwide. About 13% of the world’s population are living with CKD; and over the last 20 years, there has been over a 40% increase in mortality rates due to CKD.^1^^,^^2^

Compared to patients without CKD, CKD stages G3a to G4 (estimated glomerular filtration rate 15–60 ml/min per 1.73 m^2^) is associated with a 2 to 3 fold increase in risk of cardiovascular disease mortality.^3^ It is estimated that about 17% to 74% of patients with CKD are nonadherent to their medications.^4^ Improvement in adherence has been identified as a key strategy to enhance cardiovascular outcomes.^5^ However, the diagnosis of nonadherence, until recently, was difficult due to the lack of objective tools in busy clinics because some subjective methods have been found to be unreliable.^6^

Chemical adherence testing (CAT) is a novel, objective, robust and clinically reliable test that uses liquid-chromatography tandem mass spectrometry to assess adherence to medications. A random spot urine or blood sample is screened to determine the presence or absence of 70 of the most common cardio-metabolic medications (Supplementary Table S1) (including antihypertensives, lipid lowering medications, and glucose lowering drugs).^7^ There are guidelines available to help implement the use of the test and address common questions about the clinical use of the test.^7^

Currently, CAT is being used in routine care in some specialist hypertension clinics across Europe and has been recommended by the European Society of Cardiology and the European Society of Hypertension as the method to be used to measure adherence in patients with suspected resistant hypertension.^8^

The use of CAT is limited outside of hypertension and to the best of our knowledge there has been no publication that has used CAT to diagnose medication nonadherence in renal patients.

The aim of this study was therefore to demonstrate and highlight the usefulness of CAT to determine the prevalence of nonadherence to cardio-metabolic medications in patients attending routine renal clinics.

## Results

In this cross-sectional study, a random spot urine sample was collected from 106 consecutive consented patients between November 2019 and March 2020 attending the renal clinic at the University Hospitals of Leicester NHS Trust. Participants were eligible if they were prescribed at least 1 oral cardio-metabolic medication. The participants were informed that their urine samples will be analyzed to confirm the presence or absence of prescribed cardio-metabolic medications. To ensure that there was no change in the behavior of participants, the urine samples were collected immediately after consent was obtained. The results from CAT were not shared with the participants in this study. The study was approved by the Local Research Ethics Committee (REC reference: 17/EM/0027) (Supplementary Methods).

The mean age of the cohort was 71.1 ± 14.1 years, 48.1% were female, 68.9% were White, mean body mass index of the cohort was 29.1 ± 5.7 kg/m^2^, mean estimated glomerular filtration rate was 38.2 ± 20.9 ml/min per 1.73 m^2^, 83.0% had hypertension, 35.8% had type 2 diabetes mellitus and the median total number of prescribed medications was 3 (2–4) (Table 1).

**Table 1:** Demographic and clinical characteristics of population

Variable	All	Adherent	Nonadherent	*P*-value
Number (%)	106 (100)	58 (55)	48 (45)	
Age (yrs [mean, SD])	71.1 (14.1)	68.0 (17.9)	76.9 (11.0)	0.0033
Female (*n*, %)	51 (48.1)	31 (53.4)	20 (41.7)	0.2269
White (*n*, %)	73 (68.9)	40 (69.0)	33 (68.8)	0.9810
BMI kg/m^2^ (mean, SD)	29.1 (5.7)	29.6 (5.5)	28.6 (5.9)	0.3694
eGFR ml/min per 1.73 m^2^ (mean, SD)	38.2 (20.9)	42.9 (23.4)	32.5 (15.9)	0.0102
Hypertension (*n*, %)	88 (83.0)	47 (81.0)	41 (85.4)	0.6110
Type 2 diabetes (*n*, %)	38 (35.8)	17 (29.3)	21 (43.8)	0.1228
Total number of prescribed medications (median, interquartile range)	3 (2–4)	2 (1–3)	4 (2–5)	<0.0001

BMI, body mass index; eGFR, estimated glomerular filtration rate.

Data are presented as number (percentage), mean (SD), median (interquartile range)

Statistical analysis by *t*-test (normally distributed continuous variables), Mann Whitney U test (nonnormally distributed continuous variables) or chi-square test (nominal variables).

Urine samples were collected from the participants. The samples were then stored at −80 °C and then batch-analyzed as we have described in a previous study.^9^ The samples were analyzed to confirm the presence or absence of 70 of the most commonly prescribed cardio-metabolic medications (Supplementary Table S1) using an Agilent 1200 series binary LC system paired with an Agilent 6490 tandem mass spectrometry operated in positive and negative electrospray ionization modes.

Patients were determined to be nonadherent if at least 1 of their prescribed cardio-metabolic medication was not detected in their urine sample. Overall, 45% of the cohort was nonadherent to at least 1 of the prescribed cardio-metabolic medications with 14.2% of the cohort found to be nonadherent to all prescribed cardio-metabolic medications (Table 1). In the cohort, 28.3%, 7.5%, 3.8%, and 5.7% of patients were found to be nonadherent to 1, 2, 3, and at least 4 cardio-metabolic medications respectively.

**Figure 1:**
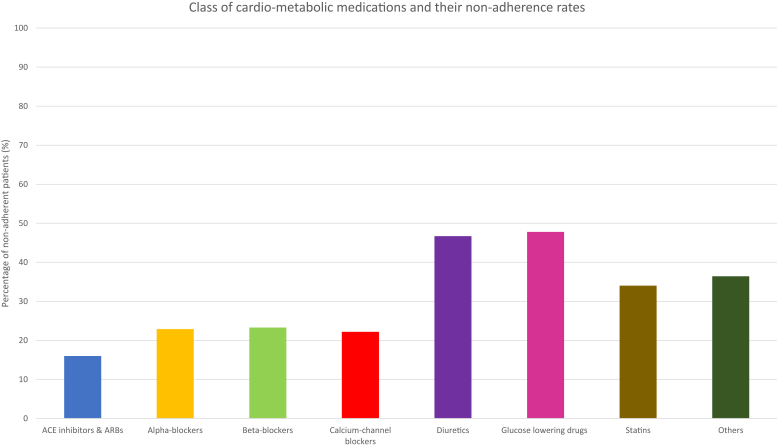
Nonadherence rates varying depending on class of cardio-metabolic medication.

Patients were most commonly nonadherent to glucose lowering drugs (11/23; 47.8%). This was followed by, in descending order of nonadherence, to diuretics (21/45; 46.7%), statins (16/47; 34.0%), beta-blockers (7/30; 23.3%), alpha-blockers (8/35; 22.9%), calcium-channel blockers (10/45; 22.2%) and angiotensin-converting enzyme inhibitors or angiotensin receptor blockers (8/50; 16.0%) (Figure 1).

Nonadherence was significantly associated with increased age (68.0 ± 17.9 years [adherent group] vs. 76.9 ± 11.0 years [nonadherent group], *P* = 0.0033), lower estimated glomerular filtration rate (42.9 ± 23.4 ml/min per 1.73 m^2^ [adherent group] vs. 32.5 ± 15.9 ml/min per 1.73 m^2^ [nonadherent group], *P* = 0.0102] and a higher total number of prescribed cardio-metabolic medications (2 [1–3] vs. 4 [2–5], *P* < 0.0001) (Table 1).

After multivariate analysis, the number of prescribed medications (odds ratio: 1.65 [95% confidence intervals 1.20-2.26], *P* = 0.002) and age (odds ratio: 1.06 [95% confidence intervals 1.02-1.11], *P* = 0.003) remained as significant predictors of nonadherence.

## Discussion

This is the first study to use CAT to diagnose nonadherence in patients recruited from renal clinics and shows that 45% are nonadherent to at least 1 medication and about 14% are nonadherent to all prescribed cardio-metabolic medications. It also demonstrated that the risk of nonadherence increases by 65% for each additional prescribed medication. This result corresponds well with previous evidence from the use of CAT in patients with hypertension, where 40% of those on 3 medications were likely to be nonadherent and it nearly doubled with every increase in the number of prescribed medications (Supplementary Reference S1). This information would be useful in clinics to identify the patients most at risk of nonadherence. Further, nonadherence was seen in a variety of medications with the highest rates of nonadherence being to glucose lowering drugs.

Out of all the classes of antihypertensive medications, the prevalence of nonadherence was highest with diuretics. The reason for this may be due to the some of the adverse effects of this class of antihypertensives, also usually thiazide and potassium-sparing diuretics are used at a later stage in hypertension management and therefore patients are more likely to be nonadherent at this stage due to the increased number of medications required to manage their condition. Loop diuretics such as furosemide tend to be used more frequently in renal patients; however, patients tend to not take this medication on the day of their clinic appointment because they may worry about needing to go to the bathroom more frequently. This precise information of the type of medication that a patient is not taking can help address their reason for nonadherence.

Currently, interventions to improve nonadherence in renal patients have not been effective in tackling this problem.^4^ This is despite the importance given in renal clinics of the close follow-up of patients and careful management of medications both to manage comorbidity and slow the progression of CKD itself. CAT may help overcome this challenge in the routine care of renal patients with cardio-metabolic disease who often are on multiple medications. Previous studies that used CAT in patients with hypertension demonstrated improvements in nonadherence and also reduced systolic blood pressure by 20 mmHg at follow-up, a reduction which would be equivalent to the effect of adding 2 antihypertensive medications (Supplementary References S2 and S3). Further, the test allows an open and nonjudgmental discussion with patients that can help address the reasons for nonadherence (Supplementary Reference S4).

It is important to note that CKD is one of the most prevalent multimorbid conditions, and as such renal patients often require multiple medications to manage their health. This can lead to polypharmacy, which is known to reduce medication adherence. A large cross-sectional study of prevalence in Scottish primary care found that close to 100% of renal patients had at least 1 comorbidity compared to about 50% of nonrenal patients (Supplementary Reference S5). This highlights the complexity of managing renal patients and the importance of strategies to improve medication adherence, such as the use of CAT. The findings of our study suggest that nonadherence to cardio-metabolic medications is a common issue among renal patients, particularly in those taking multiple medications and have a lower estimated glomerular filtration rate. These findings can inform the development of targeted interventions to improve medication adherence and ultimately improve patient outcomes in this high-risk population.

The application of CAT in renal patients present distinct challenges and considerations compared to other contexts. Polypharmacy and the complex medication regimens in these patients may impact how multiple medications interact, whereas altered pharmacokinetics due to impaired kidney function could affect CAT accuracy because there could be a longer duration of detection in urine in this cohort of patients and therefore less nonadherence may be detected. The dynamic nature of management and changing prescriptions, along with the higher comorbidity burden, require nuanced interpretation of CAT results. Engaging renal patients effectively and discussing CAT outcomes in light of long-term management demands tailored approaches.

Our study has limitations because it is a single-center observational study. We do not have data on the total number of patients who were approached for consent or the total number of patients that declined to take part in the study. In addition, because patients were recruited on attending the renal clinic, the participants may not represent the wider population of renal patients. CAT provides only a snapshot of adherence because medications can be detected for about 4 to 6 half-lives. Therefore, it is possible that if a patient had omitted a loop diuretic such as furosemide on the day of the clinic visit, the medication would not be detected. Conversely, if a patient starts to adhere to their medications close to the date of their appointment at the clinic (also referred to as ‘white coat adherence’), the rates of nonadherence could be underestimated (Supplementary Reference S6). Another potential limitation to consider is the effect that pharmacokinetics may have especially in renal patients, but we would anticipate that the excretion of medications in urine would be prolonged and if anything, the nonadherence rates would have been underestimated.

Despite these limitations, this study is novel and used objective measures to assess adherence; and highlights the usefulness of CAT as a clinical tool that can provide a fresh impetus in tackling the problem of nonadherence to cardio-metabolic medications in renal patients. Larger studies, including intervention studies, are needed to develop robustness in the use of CAT.

## Conclusion

In conclusion, this is the first study that used CAT in a single spot urine sample to diagnose nonadherence in patients recruited from renal clinics. It found that nearly half of renal patients were nonadherent to at least 1 of their prescribed cardio-metabolic medications and the nonadherence rates increased by 65% for every increase in the number of prescribed cardio-metabolic medications. These findings highlight the utility and importance of the use of CAT to diagnose nonadherence in renal patients on cardio-metabolic medications.

## Disclosure

All the authors declared no competing interests.

## Supplementary Material

**Supplementary Methods.**

**Supplementary References.**

**Table S1.** List of 70 commonly prescribed cardio-metabolic medications.

**STROBE Checklist.**
