# Supplementary material for: An Innovative Chemical Adherence Test Demonstrates Very High Rates of Nonadherence to Oral Cardio-Metabolic Medications
Source: Kidney Int Rep. 2023 Sep 29;8(12):2818–21. doi: 10.1016/j.ekir.2023.09.033 (PMC10719591; doi:10.1016/j.ekir.2023.09.033)
Supplement: Supplementary File (PDF) [file mmc1.pdf]

Supplementary Materials

Supplementary information is available at KI Report's website

- STROBE Checklist
- Supplementary Methods
- Supplementary References
- Supplementary Table S1. List of 70 commonly prescribed cardio-metabolic medications.

STROBE Statement—checklist of items that should be included in reports of observational studies

	Item No.	Recommendation	Page No.	Relevant text from manuscript
**Title and abstract**	1	(*a*) Indicate the study's design with a commonly used term in the title or the abstract	1	Lines (1-2); An innovative chemical adherence test demonstrates very high rates of non-adherence to oral cardio-metabolic medications
		(*b*) Provide in the abstract an informative and balanced summary of what was done and what was found	N/A	No abstract required as this is a research letter
**Introduction**				
Background/rationale	2	Explain the scientific background and rationale for the investigation being reported	2	Introduction (Lines 28-53)
Objectives	3	State specific objectives, including any prespecified hypotheses	2	Lines (51-53); The aim of this study was therefore to demonstrate and highlight the usefulness of CAT to determine the prevalence of non-adherence to cardio-metabolic medications in patients with CKD attending routine renal clinics.
**Methods**				
Study design	4	Present key elements of study design early in the paper	2	Lines (55-57); In this cross-sectional study, a random spot urine sample was collected from 106 consecutive

				consented patients with CKD between November 2019 and March 2020 attending the renal clinic at the University Hospitals of Leicester NHS Trust.
Setting	5	Describe the setting, locations, and relevant dates, including periods of recruitment, exposure, follow-up, and data collection	2-3 and Page 12 of Supplementary Materials	Results (Lines 54-63) & Supplementary Methods (Lines 17-36)
Participants	6	(*a*) *Cohort study*—Give the eligibility criteria, and the sources and methods of selection of participants. Describe methods of follow-up<br>*Case-control study*—Give the eligibility criteria, and the sources and methods of case ascertainment and control selection. Give the rationale for the choice of cases and controls<br>*Cross-sectional study*—Give the eligibility criteria, and the sources and methods of selection of participants	2	Lines (55-57); In this cross-sectional study, a random spot urine sample was collected from 106 consecutive consented patients with CKD between November 2019 and March 2020 attending the renal clinic at the University Hospitals of Leicester NHS Trust.
		(*b*) *Cohort study*—For matched studies, give matching criteria and number of exposed and unexposed<br>*Case-control study*—For matched studies, give matching criteria and the number of controls per case	N/A	This was a cross-sectional study
Variables	7	Clearly define all outcomes, exposures, predictors, potential confounders, and effect modifiers. Give diagnostic criteria, if applicable	Page 12 of Supplementary Materials	Lines (18-21); Data on demographic variables were collected from the patient renal clinic letters. The demographic variables for which data was collected included age, sex, ethnicity, total number of prescribed medications,

				presence of specified co-morbidities (diabetes and hypertension), body mass index (BMI) and estimated glomerular filtration rate (eGFR).
Data sources/ measurement	8*	For each variable of interest, give sources of data and details of methods of assessment (measurement). Describe comparability of assessment methods if there is more than one group	3 and Page 12 of Supplementary Materials	Results (Lines 68-72) & Supplementary Methods (Lines 22-25)
Bias	9	Describe any efforts to address potential sources of bias	3	Lines (59-61); To ensure that there was no change in the behaviour of patients, the urine samples were collected immediately after consent was obtained.
Study size	10	Explain how the study size was arrived at	N/A	No sample size calculation was carried out.

Continued on next page

Quantitative variables	11	Explain how quantitative variables were handled in the analyses. If applicable, describe which groupings were chosen and why Page 12 of Supplementary Materials	Lines (26-32); Analysis of quantitative variables	
Statistical methods	12	*(a)* Describe all statistical methods, including those used to control for confounding Page 12 of Supplementary Materials	Lines (26-36); Statistical analysis	
		(b) Describe any methods used to examine subgroups and interactions N/A There were no sub-groups		
		(c) Explain how missing data were addressed N/A There was no missing data		
		(*d*) *Cohort study*—If applicable, explain how loss to follow-up was addressed<br>*Case-control study*—If applicable, explain how matching of cases and controls was addressed<br>*Cross-sectional study*—If applicable, describe analytical methods taking account of sampling strategy	2	Lines (55-57); In this cross-sectional study, a random spot urine sample was collected from 106 consecutive consented patients with CKD between November 2019 and March 2020 attending the renal clinic at the University Hospitals of Leicester NHS Trust.
		(*e*) Describe any sensitivity analyses N/A		
**Results**				
Participants	13*	(a) Report numbers of individuals at each stage of study—eg numbers potentially eligible, examined for eligibility, confirmed eligible, included in the study, completing follow-up, and analysed	N/A	This was a cross-sectional study with only one stage
		(b) Give reasons for non-participation at each stage	N/A	

		(c) Consider use of a flow diagram	N/A	
Descriptive data	14*	(a) Give characteristics of study participants (eg demographic, clinical, social) and information on exposures and potential confounders	Page 3	Lines (64-67); The mean age of the cohort was 71.1 ± 14.1 years, 48.1% were female, 68.9% were White, mean BMI of the cohort was 29.1 ± 5.7 kg/m2, mean eGFR was 38.2 ± 20.9 ml/min/1.73 m2, 83.0% had hypertension, 35.8% had type 2 diabetes and the median total number of prescribed medications was 3 (2-4) (Table 1).
		(b) Indicate number of participants with missing data for each variable of interest	N/A	There was no missing data
		(c) *Cohort study*—Summarise follow-up time (eg, average and total amount)	N/A	This was a cross-sectional study
Outcome data	15*	*Cohort study*—Report numbers of outcome events or summary measures over time	N/A	This was a cross-sectional study
		Case-control study—Report numbers in each exposure category, or summary measures of exposure	N/A	This was a cross-sectional study
		Cross-sectional study—Report numbers of outcome events or summary measures	Page 3	Lines (73-83); Patients were determined to be non-adherent if at least one of their prescribed cardio-metabolic medication was not detected in their urine sample. Overall, 45% of the cohort was non-adherent to at least one of the prescribed cardio-metabolic medications with 14.2% of the cohort found to be non-adherent to all prescribed cardio-metabolic medications (Table 1). In the cohort; 28.3%, 7.5%, 3.8% and 5.7% of patients were found to be non-adherent to one, two, three

				and at least four cardio-metabolic medications respectively.<br><br>Patients were most commonly non-adherent to GLDs (N = 11/23; 47.8%). This was followed by in descending order of non-adherence to: diuretics (N = 21/45; 46.7%), statins (N = 16/47; 34.0%), beta-blockers (N = 7/30; 23.3%), alpha-blockers (N = 8/35; 22.9%), calcium-channel blockers (CCBs) (N = 10/45; 22.2%) and angiotensin-converting enzyme (ACE) inhibitors or angiotensin receptor blockers (ARBs) (N = 8/50; 16.0%) [Figure 1].
Main results	16	(*a*) Give unadjusted estimates and, if applicable, confounder-adjusted estimates and their precision (eg, 95% confidence interval). Make clear which confounders were adjusted for and why they were included	Page 3	Lines (84-90); Non-adherence was significantly associated with increased age [68.0 ± 17.9 years (adherent group) vs. 76.9 ± 11.0 years (non-adherent group), P = 0.0033], lower eGFR [42.9 ± 23.4 ml/min/1.73 m2 (adherent group) vs. 32.5 ± 15.9 ml/min/1.73 m2 (non-adherent group), P = 0.0102] and a higher total number of prescribed cardio-metabolic medications [2 (1-3) {adherent group} vs. 4 (2-5) {non-adherent group}, P < 0.0001] (Table 1).<br><br>After multivariate analysis, the number of prescribed medications [odds ratio: 1.65 (95% confidence

			intervals 1.20-2.26), P = 0.002] and age [odds ratio: 1.06 (95% confidence intervals 1.02-1.11), P = 0.003] remained as significant predictors of non-adherence.
	(*b*) Report category boundaries when continuous variables were categorized	Page 3	Lines (84-90); Non-adherence was significantly associated with increased age [68.0 ± 17.9 years (adherent group) vs. 76.9 ± 11.0 years (non-adherent group), P = 0.0033], lower eGFR [42.9 ± 23.4 ml/min/1.73 m2 (adherent group) vs. 32.5 ± 15.9 ml/min/1.73 m2 (non-adherent group), P = 0.0102] and a higher total number of prescribed cardio-metabolic medications [2 (1-3) {adherent group} vs. 4 (2-5) {non-adherent group}, P < 0.0001] (Table 1).<br><br>After multivariate analysis, the number of prescribed medications [odds ratio: 1.65 (95% confidence intervals 1.20-2.26), P = 0.002] and age [odds ratio: 1.06 (95% confidence intervals 1.02-1.11), P = 0.003] remained as significant predictors of non-adherence.
	(*c*) If relevant, consider translating estimates of relative risk into absolute risk for a meaningful time period	N/A	There were no estimates of relative risk

Continued on next page

Other analyses	17	Report other analyses done—eg analyses of subgroups and interactions, and sensitivity analyses	N/A	There were no sub-groups
**Discussion**				
Key results	18	Summarise key results with reference to study objectives	Pages 4-5	Lines (91-153); Discussion
Limitations	19	Discuss limitations of the study, taking into account sources of potential bias or imprecision. Discuss both direction and magnitude of any potential bias	Page 5	Lines (138-149); Our study has limitations as it is a single-centre observational study. We do not have data on the total number of patients who were approached for consent or the total number of patients that declined to take part in the study. Additionally as patients were recruited on attending the renal clinic, the participants may not represent the wider population of renal patients. CAT provides only a snap shot of adherence as medications can be detected for around 4-6 half lives. Thus, it is possible that if a patient had omitted a loop diuretic such as furosemide on the day of the clinic visit, the medication would not be detected. Conversely, if patient start to adhere to their medications around the date of their appointment at the clinic (also referred to as 'white coat adherence'), the rates of non-adherence could be underestimated S6. Another potential limitation to consider is

				the effect that pharmacokinetics may have especially in renal patients but we would anticipate that the excretion of medications in urine would be prolonged and if anything the non-adherence rates would have been underestimated.
Interpretation	20	Give a cautious overall interpretation of results considering objectives, limitations, multiplicity of analyses, results from similar studies, and other relevant evidence	Pages 4-5	Lines (91-153); Discussion
Generalisability	21	Discuss the generalisability (external validity) of the study results	Page 5	Lines (150-153); Despite these limitations, this study is novel and used objective measures to assess adherence, and highlights the usefulness of CAT as a clinical tool that can provide a fresh impetus in tackling the problem of non-adherence to cardio-metabolic medications in renal patients. Larger studies, including intervention studies are needed to develop robustness in the use of CAT.
**Other information**				
Funding	22	Give the source of funding and the role of the funders for the present study and, if applicable, for the original study on which the present article is based	Page 6	Lines (164-167); HO is funded by Servier Affaires Medicale. The views expressed are those of the author(s) and not necessarily those of Servier Affaires Medicale.<br>SS, KK & PG are supported by the National Institute for Health

Research (NIHR) Applied Research Collaboration East Midlands (ARC EM) and the NIHR Leicester Biomedical Research Centre (BRC).

*Give information separately for cases and controls in case-control studies and, if applicable, for exposed and unexposed groups in cohort and cross-sectional studies.

Note: An Explanation and Elaboration article discusses each checklist item and gives methodological background and published examples of transparent reporting. The STROBE checklist is best used in conjunction with this article (freely available on the Web sites of PLoS Medicine at http://www.plosmedicine.org/, Annals of Internal Medicine at http://www.annals.org/, and Epidemiology at http://www.epidem.com/). Information on the STROBE Initiative is available at www.strobe-statement.org.

Supplementary Methods

Data on demographic variables were collected from the patient renal clinic letters. The demographic variables for which data was collected included age, sex, ethnicity, total number of prescribed medications, presence of specified co-morbidities (diabetes and hypertension), body mass index (BMI) and estimated glomerular filtration rate (eGFR).

LC-MS/MS was used to determine the presence or absence of a range of 70 common cardio-metabolic medications including antihypertensives, lipid lowering medications and medications used for diabetes (Supplementary Table S1). Patients were determined to be non-adherent if at least one of their prescribed cardio-metabolic medication was not detected in their urine sample.

SPSS version 28 for Windows was used to carry out the statistical analysis. The parametric continuous variables were presented as means with standard deviations, the non-parametric continuous variables were presented as medians with interquartile ranges and the categorical variables were presented as percentages. Chi-squared test was used to determine the differences between the adherent and non-adherent cohorts for the categorical variables. Student's t-test was used for the normally distributed continuous variables. Mann Whitney U test was used for the non-normally distributed continuous variables.

A stepwise logistic regression analysis was performed between the adherence groups and the following independent variables: age, eGFR and the total number of prescribed medications. Variables with a p value below 0.05 were included in a stepwise logistic regression analysis with non-adherence as the dependent variable.

Supplementary References

S1. Gupta P, Patel P, Štrauch B, et al. Risk Factors for Nonadherence to Antihypertensive Treatment. Hypertension. Jun 2017;69(6):1113-1120. doi:10.1161/hypertensionaha.116.08729

S2. Gupta P, Patel P, Štrauch B, et al. Biochemical Screening for Nonadherence Is Associated With Blood Pressure Reduction and Improvement in Adherence. Hypertension. Nov 2017;70(5):1042-1048. doi:10.1161/hypertensionaha.117.09631

S3. Brinker S, Pandey A, Ayers C, et al. Therapeutic drug monitoring facilitates blood pressure control in resistant hypertension. *J Am Coll Cardiol*. Mar 4 2014;63(8):834-5. doi:10.1016/j.jacc.2013.10.067

S4. Gupta P, Patel P, Horne R, Buchanan H, Williams B, Tomaszewski M. How to screen for non-adherence to antihypertensive therapy. *Current hypertension reports*. 2016;18(12):1-8.

S5. MacRae C, Mercer SW, Guthrie B, Henderson D. Comorbidity in chronic kidney disease: a large cross-sectional study of prevalence in Scottish primary care. British Journal of General Practice. 2021;71(704):e243-e249. doi:10.3399/bjgp20X714125

S6. Chatterjee JS. From compliance to concordance in diabetes. J Med Ethics. Sep 2006;32(9):507-10. doi:10.1136/jme.2005.012138

Supplementary Table S1. List of 70 commonly prescribed cardio-metabolic medications.

Alfuzosin	Alogliptin	Amiloride	Amlodipine	Apixaban
Atenolol	Atorvastatin	Bendroflumethiazide	Bisoprolol	Bumetanide
Canagliflozin	Candesartan	Carvedilol	Clonidine	Clopidogrel
Dabigatran	Dapagliflozin	Diltiazem	Doxazosin	Edoxaban
Empagliflozin	Enalapril	Eplerenone	Felodipine	Fluvastatin
Furosemide	Glibenclamide	Gliclazide	Glimepiride	Glipizide
Hydrochlorothiazide	Indapamide	Irbesartan	Ivabradine	Labetalol
Lacidipine	Lercanidipine	Linagliptin	Lisinopril	Losartan
Metformin	Metoprolol	Minoxidil	Moxonidine	Nateglinide
Nebivolol	Nicardipine	Nicorandil	Nifedipine	Olmesartan
Perindopril	Pioglitazone	Pravastatin	Prasugrel	Prazosin
Propranolol	Ramipril	Ranolazine	Repaglinide	Rivaroxaban
Rosuvastatin	Saxagliptin	Sitagliptin	Spironolactone	Telmisartan
Ticagrelor	Trandolapril	Triamterene	Valsartan	Verapamil
